# Monitoring HIV testing and pre-exposure prophylaxis information seeking by combining digital and traditional data

**DOI:** 10.1186/s12879-021-05907-0

**Published:** 2021-02-25

**Authors:** Derek C. Johnson, Alicia L. Nobles, Theodore L. Caputi, Michael Liu, Eric C. Leas, Steffanie A. Strathdee, Davey M. Smith, John W. Ayers

**Affiliations:** 1 Department of Medicine, Division of Infectious Diseases and Global Public Health, University of California San Diego, 9500 Gilman Dr, La Jolla, California 92093 USA; 2 The Center for Data Driven Health at the Qualcomm Institute, University of California San Diego, La Jolla, California USA; 3 Department of Health Sciences, University of York, York, UK; 4 University of Oxford, Oxford, UK; 5 Department of Family Medicine and Public Health, Division of Health Policy, University of California San Diego, La Jolla, California USA

**Keywords:** Google trends, HIV, PrEP, Internet, HIV testing

## Abstract

**Background:**

Public health is increasingly turning to non-traditional digital data to inform HIV prevention and control strategies. We demonstrate a parsimonious method using both traditional survey and internet search histories to provide new insights into HIV testing and pre-exposure prophylaxis (PrEP) information seeking that can be easily extended to other settings.

**Method:**

We modeled how US internet search volumes from 2019 for HIV testing and PrEP compared against expected search volumes for HIV testing and PrEP using state HIV prevalence and socioeconomic characteristics as predictors. States with search volumes outside the upper and lower bound confidence interval were labeled as either over or under performing. State performance was evaluated by (a) Centers for Disease Control and Prevention designation as a hotspot for new HIV diagnoses (b) expanding Medicaid coverage.

**Results:**

Ten states over-performed in models assessing information seeking for HIV testing, while eleven states under-performed. Thirteen states over-performed in models assessing internet searches for PrEP information, while thirteen states under-performed. States that expanded Medicaid coverage were more likely to over perform in PrEP models than states that did not expand Medicaid coverage. While states that were hotspots for new HIV diagnoses were more likely to over perform on HIV testing searches.

**Conclusion:**

Our study derived a method of measuring HIV and PrEP information seeking that is comparable across states. Several states exhibited information seeking for PrEP and HIV testing that deviated from model assessments. Statewide search volume for PrEP information was affected by a state’s decision to expand Medicaid coverage. Our research provides health officials with an innovative way to monitor statewide interest in PrEP and HIV testing using a metric for information-seeking that is comparable across states.

## Background

As people increasingly turn to digital sources of news and information, online activity has the potential to become a window into the public’s consciousness [1]. Measuring the public’s online information seeking has the potential to predict health behavior, as what people are searching the internet for can be predictive of what they intend to do in the future [2]. It is possible that seeking information about HIV testing and Pre-Exposure Prophylaxis (PrEP) online could be a new surveillance tool in the fight against HIV. Previous studies have shown a spike in internet searches for HIV testing has corresponded with increases in HIV testing, suggesting that seeking HIV testing information online could be predictive of testing behavior [3]. Utilizing internet searches could be a way to enhance the surveillance of the public’s interest in seeking information on HIV and HIV health seeking behavior. Past efforts to enhance HIV surveillance relied mostly on upscaling traditional data (e.g., clinical records or surveys) that have intrinsic shortcomings, such as a limited ability to provide current information. For instance, the most recent data for HIV testing on AIDSvu.org is from 2016 and the most recent AIDSvu.org data on PrEP usage is from 2018 [4]. These limitations have driven public health to increasingly turn to digital data, such as news, social media, and internet searches, to learn how people seek HIV information [5–8]. For example, internet search trends can be used to investigate public interests as evident by actor Charlie Sheen’s HIV positive disclosure concurring with record levels of Google searches for HIV awareness, HIV testing, and condoms [3]. This finding was valid, as it was later confirmed by traditional data after a 16 month delay [7]. Internet search histories have potential utility for assessing both help-seeking behavior regarding public interest in PrEP for HIV prevention and for HIV testing. For example, one study conducted in Hong Kong found that a direct relation between HIV news trends and online search behavior for issues regarding HIV/AIDS and men who have sex with men (MSM) [9]. Other studies have found that areas with high levels of HIV prevalence have greater internet search volumes for HIV related terms then areas of low HIV prevalence [10]. These studies show that the use of internet search histories combined with traditional surveillance data has the potential to create synergies that can yield new insights into HIV related health behavior.

Our study methods use both internet search histories and traditional survey data to provide new insights on information seeking for HIV testing and PrEP information that can be easily replicated and extended to other settings and outcomes. Specifically, we predicted expected internet search volumes for HIV testing and PrEP based on statewide HIV prevalence and socioeconomic (SES) factors and compared them to observed search volumes in a model that allows us to identify if US states over or under perform against expectations. Moreover, we evaluated how state performance varied by (a) states that are designated as hotspots for new HIV diagnoses (b) states that received Medicaid expansion funding.

## Methods

Our study used data from multiple sources. 1) We obtained the most current state-level prevalence of HIV from the Center for Disease Control and Prevention HIV surveillance report [11], which is from 2018. HIV prevalence was chosen over HIV incidence because we were interested in look at the association between the total number of HIV cases in a state and internet searches for HIV testing and PrEP 2) The following state-level socioeconomic attributes we obtained from the 2018 Center for Disease Control and Prevention’s Behavioral Risk Factor Surveillance System (BRFSS): proportion of males, white non-Hispanics, people aged 45 years or older, and people with household income over $50,000 [12]. 3) We obtained 2019 state-level annual internet search volumes for HIV testing and PrEP from using the Google Trends API. Information available through this API includes the volume of searches for each term, the number of searches per unit of time, and the geographic location of the searches (country, region, state, city, metropolitan area). Search volume data was calculated as a query fraction of the proportion of searches of a specific search term relative to all searches measured per 10 million searches. Standardizing search volumes was done in order to account for population sizes. We defined HIV testing searches as any query that included the terms “HIV” and “test,” “tests,” or “testing”, “AIDS test”, or “oraquick”. We defined PrEP searches as any query that included the terms “PrEP” and “HIV” or “pre-exposure prophylaxis HIV” or “Truvada” or “Descovy”.

Internet search volumes are withheld by Google for states where searches do not achieve a minimum threshold of searches. As a result, we could not obtain search data for HIV testing for five states (Alaska, Montana, South Dakota, Vermont, and Wyoming). PrEP search data could not be obtained for two states (Vermont and Wyoming). 4) We obtained data on states that expanded Medicaid coverage from the Kaiser Family Foundation [13].

Our analysis followed a four-step process. First, we fit Poisson regression models with state-level HIV prevalence data and state-level socioeconomic attributes to predict the expected internet search volumes of HIV testing and PrEP for each state. Second, we fit a centered least squares regression line of expected search volumes from our models versus observed search volumes from Google Trends. Third, we compared the expected search volumes from our models in step one with the observed search volumes from Google Trends for each state in order to assess the level of information seeking for HIV testing and PrEP by calculating the percent difference between the observed and expected values of the centered least squares regression (i.e., (observed-fitted)/fitted * 100%).

**Fig. 1:**
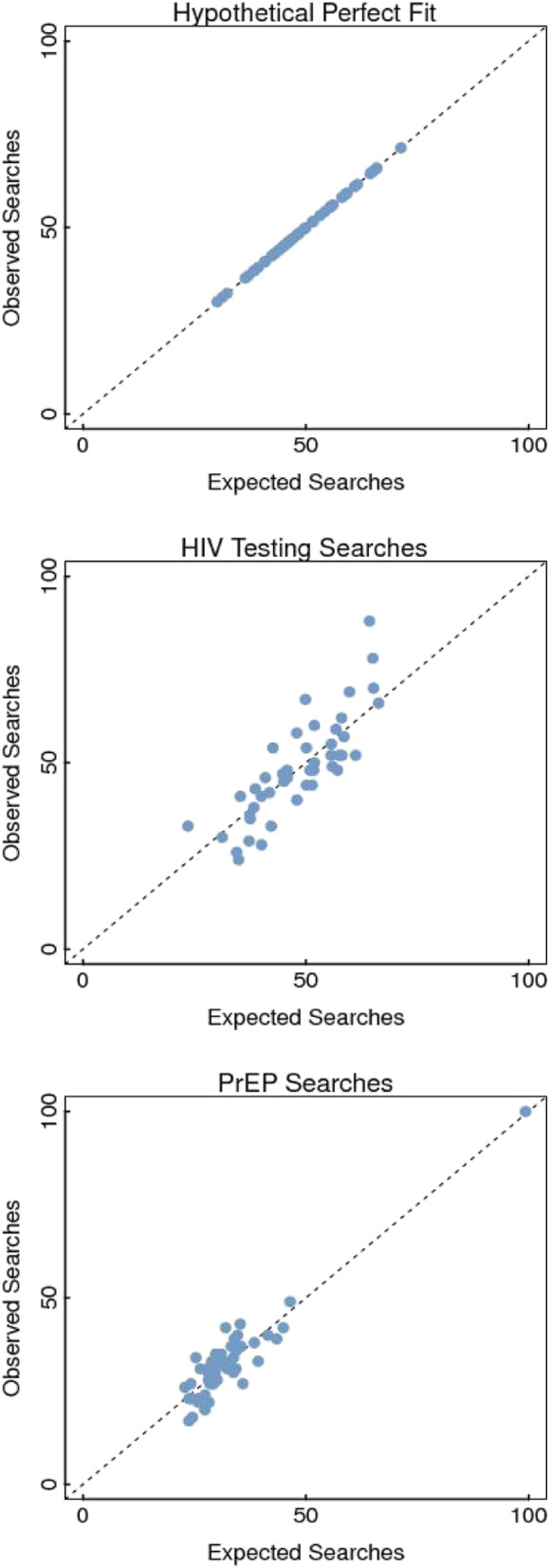
Observed vs. Expected HIV Testing and PrEP Internet Searches as Compared to a Hypothetical Perfectly Fitting Regression Line

States with observed information seeking (measured by observed internet search volumes for HIV testing and PrEP) greater than their expected information seeking (predicted internet search volumes by the Poisson regression model) were considered to be over performing and exhibit greater information seeking for HIV testing and PrEP than expected given their prevalence of HIV. States that were over performing above the 95% confidence interval were highlighted in our results (see example of plotting observed vs. expected observations in Fig. 1). Similarly, states with observed information seeking less than their expected information seeking were considered to be underperforming and exhibit less information seeking for HIV testing and/or PrEP than expected given their prevalence of HIV. States that were under performing below the 95% confidence interval were highlighted in our results To describe statistical uncertainty between expected and observed search volumes, we used bootstrap sampling to calculate the 95% confidence interval (CI) for the regression line and labeled observations outside the confidence band as states that over or under performed.

Fourth, to understand which states typically under or over performed we contrasted the deviations in expected searches against (a) What states were designated as hotspots for new HIV diagnoses by the Centers for Disease Control and Prevention (CDC) [11], (b) What states received Medicaid expansion funding that covered HIV testing and PrEP [13].

## Results

We observed different levels of information seeking for HIV testing and PrEP across states **(**Fig. 1**)**. Ten states over-performed for HIV testing searches. Georgia exhibited the greatest difference with 36.8% more searches than expected followed closely by Rhode Island (35.2%), then Indiana (28.8%), Pennsylvania (22.9%), Nevada (18.6%), Florida (18.0%), Louisiana (17.3%), Washington (15.3%), Iowa (13.4%), and Virginia (10.0%) **(**Table 1**)**. Conversely, eleven states under-performed for HIV testing. New Hampshire exhibited the greatest difference with − 34.1% less searches than expected, followed by Maine (− 32.2%), Idaho (− 26.1%), Nebraska (− 21.9%), Oregon (− 20.5%), New Mexico (− 18.5%), Mississippi (− 16.8%), Arkansas (− 15.7%), Alabama (− 13.6%), Massachusetts (− 12.4%), Arizona (− 10.3%),

**Table 1:** Statewide Proportional Differences Between Observed and Expected Internet Search Volumes for Information Seeking About HIV testing and PrEP

U.S State	HIV prevalence per 100,000 individuals	HIV Search Differences	PrEP Search Differences	Expanded Medicaid Coverage^@^	Designated Hotspot for New HIV Infections
Alabama	394	−13.6*	−6.4	Y	N
Alaska	136.6	NA	−2.3	Y	N
Arizona	326.6	−10.3*	14.1^#^	Y	N
Arkansas	278.7	−15.7*	−1.6	Y	N
California	451.9	−9.3	4.8	Y	Y
Colorado	305	7	10.7^#^	Y	N
Connecticut	371.9	−2.9	4.1	Y	N
Delaware	461.4	−5.6	−7.7*	Y	N
District of Columbia	2515.5	−10.5	−1.9	Y	Y
Florida	691.8	18^#^	−5.3	N	Y
Georgia	745.6	36.9^#^	0.4	N	Y
Hawaii	229.4	−11.3	−1.1	Y	N
Idaho	94.1	−26.1*	− 31*	N	N
Illinois	384.7	−5.0	12.6^#^	Y	Y
Indiana	248.6	28.8^#^	−5.0	Y	Y
Iowa	128.5	13.5^#^	−17.2*	Y	N
Kansas	154.6	−0.7	−13.3*	N	N
Kentucky	239.2	1.0	10.2^#^	Y	Y
Louisiana	654.4	17.3^#^	3.9	Y	Y
Maine	158	−32.2*	11.5	N	N
Maryland	723.8	6.7	−5.2	Y	Y
Massachusetts	385.2	−12.4*	23.6^#^	Y	Y
Michigan	223.1	5.4	−9.4*	Y	Y
Minnesota	209.9	−3.5	−5.0	Y	N
Mississippi	456.9	−16.8*	0.1	N	N
Missouri	281.9	1.7	−6.1*	N	N
Montana	83.7	NA	−28.2*	Y	N
Nebraska	161.3	−21.9*	16.9^#^	Y	N
Nevada	501.4	18.6^#^	18^#^	Y	N
New Hampshire	119	−34.1*	−23.6*	Y	N
New Jersey	511.3	−2.6	−15.9*	Y	Y
New Mexico	239.9	−18.5*	−1.6	Y	N
New York	822.7	0.5	12.1^#^	Y	Y
North Carolina	411.4	−5.8	−8.4*	N	Y
North Dakota^^^	101.0	21.6	12.8	Y	N
Ohio	270.1	5.4	0.1	Y	Y
Oklahoma	229.2	2.3	−14.1*	N	N
Oregon	227	−20.5*	12.4^#^	Y	N
Pennsylvania	361.8	22.9^#^	17.3^#^	Y	Y
Rhode Island	318.4	35.2^#^	29.2^#^	Y	N
South Carolina	480.8	6.7	−23.1*	Y	N
South Dakota	104.9	NA	7.7	N	N
Tennessee	352.1	−4.3	2.8	Y	Y
Texas	471.3	7	−9.3	Y	Y
Utah	139.7	−3.4	−2.9	N	N
Vermont	149.9	NA	NA	Y	N
Virginia	365.9	10^#^	−6.4	N	N
Washington	245	15.3^#^	19.9^#^	Y	Y
West Virginia	146.7	8.4	30.7^#^	Y	N
Wisconsin	148.3	−7.9	−27.9*	N	N
Wyoming^^^	83.5	NA	NA	N	N

^#^Over performed in search models

*Underperformed in search models

^@^State decisions on the Affordable Care Act’s Medicaid expansion

^^^HIV prevalence for 2018 was missing for Wyoming and North Dakota and was substituted with HIV prevalence from 2017

Thirteen states over-performed for PrEP searches **(**Table 1**).** West Virginia exhibited the greatest difference with 30.7% more searches than expected, followed by Rhode Island (29.2%), Massachusetts (23.6%), Washington (19.9%), Nevada (18.0%), Pennsylvania (17.3%), Nebraska (16.9%), Arizona (14.1%), Illinois (12.6%), Oregon (12.4%), New York (12.1%), Colorado (10.7%), and Kentucky (10.2%). Conversely, thirteen states under-performed for PrEP searches. Idaho exhibited the greatest difference with 30.9.% less searches than expected, followed by Montana (− 28.2%), Wisconsin (− 27.9%), New Hampshire (− 23.6%), South Carolina (− 23.1%), Iowa (− 17.2%), New Jersey (− 15.9%), Oklahoma (− 14.1%), Kansas (− 13.3%), Michigan (− 9.4%), North Carolina (− 8.4%), Delaware (− 7.7%), and Missouri (− 6.1%).

States that over or under performed on HIV testing searches did not necessarily do likewise for PrEP searches (r = 0.12). For instance, Nebraska ranked 7th for excess HIV testing searches, but then ranked 43rd for PrEP searches. Four states (Washington, Nevada, Pennsylvania, and Rhode Island) over-performed for both HIV testing and PrEP searches, while only 2 states (New Hampshire and Idaho) under-performed for both. States that expanded Medicaid coverage were more likely to over perform more on PrEP searches compared to states that did not expand Medicaid coverage (z = 2.04, *p* < 0.041). States that were hotspots for new HIV diagnoses were more likely to over perform on HIV testing searches than states that were not hotspots for new HIV diagnoses (z = 2.08, *p* < 0.037).

## Discussion

Our study derived a method of measuring HIV testing and PrEP information seeking that is comparable across states. Several states exhibited information seeking for PrEP and HIV testing that deviated from what was expected in our models. A state’s performance in our models was not affected by its designation as a hotspot for new HIV infections. However, performance for PrEP information seeking was associated with a state’s decision to expand Medicaid coverage. By integrating internet search histories and traditional survey data, our results provide baseline benchmarks for monitoring statewide interest in seeking information on HIV testing and PrEP.

Our research demonstrates a need for increased access to PrEP information, particularly among states that have not expanded their Medicaid coverage. Lower interest in seeking information on PrEP for states that did not expand Medicaid coverage could be detrimental to increasing PrEP utilization given that insurance coverage affects PrEP uptake [14, 15]. Approximately 12% of PrEP users receive PrEP through Medicaid [16] and the refusal to extend coverage could deny people the ability to access PrEP. Our results, coupled with the inability to utilize PrEP due to a lack of health insurance, is a potentially disastrous combination that could result in an increase in HIV prevalence in states that underperformed in our PrEP models.

Underperformance in PrEP models could be due to the unequal distribution of PrEP across different genders, ages, and states. Our models control for age, sex, race, and income at the state level using BRFSS data. However, our models do not adjust for disparities in the distribution of PrEP. It is possible that PrEP interventions that do not specifically target key populations with indications for PrEP use could result in these neglected populations not searching for PrEP information on line, which would result in an underperformance in our PrEP models. For example, five states represented 50% of PrEP prescriptions and although women represent almost 20% of new HIV infections, they represented only 7% of PrEP prescriptions [16, 17]. These types of underlying disparities in PrEP distribution could possibly be factors influencing how people look for information on PrEP.

Our research suggest the possibility that increased attention to HIV testing, promoted by a state being listed as a CDC hotspot for new HIV diagnoses, does in fact result in increased public interest in seeking HIV testing information [11]. States that are listed as a hotspot for new HIV infections receive a rapid infusion of additional resources, expertise, and technology to develop and implement locally tailored HIV interventions [18]. It is possible that the increased promotion of HIV interventions results in more public interest in seeking HIV testing information. This would explain why states that were listed as hotspots for new HIV diagnoses were more likely to over perform on HIV testing searches than states that were not hotspots. Our results support providing states with more resources to promote HIV testing, given that our models suggest increases in searches for HIV testing are correlated with more CDC support for HIV programs.

Our study benefits from several strengths. We use a nationally representative survey to control for several SES covariates, ensuring that the US population is accurately represented. Because our methods adjusted for baseline state level SES characteristics, leaders in each individual state can use these methods to evaluate their state-specific progress. Our internet search volume data is measured in real-time, and while we used annual estimates, it is possible to use the same method to estimate weekly or monthly search volumes. Most importantly, our research presents a new method for surveillance and performance monitoring in HIV prevention.

Our research is not without limitations. Internet search volume data is aggregated and is susceptible to ecological confounding. Additionally, it cannot be used to determine which racial/ethnic, gender, or age groups are or are not engaged with HIV testing or PrEP. While it is possible that adding more search terms could affect our results, the effects of adding additional search terms to our models diminishes after the most common search terms are added, as these terms make up the vast majority of search terms that the public uses. To insure we were using the most common search terms for HIV testing and PrEP, we consulted with HIV experts on HIV testing and PrEP nomenclature. Using search data may be subject to selection bias, as not all people access the Internet equally and although some queries may reflect general curiosity rather than treatment-seeking, it is well known that internet search trends mirror many health-related behaviors [1].

## Conclusions

Our results are a call-to-action for underperforming states whose populations are not engaged in searching for information on HIV testing and PrEP. Our research provides health officials with an innovative way to monitor statewide interest in PrEP and HIV testing by highlighting the states that demonstrate the least online information seeking, which is critical for the promotion of HIV testing and PrEP as a way to help end the HIV epidemic. Further research should examine why certain states are deficient, and policy makers in deficient states should make efforts to expand HIV testing and PrEP promotion, perhaps by replicating the interventions and policies of better-performing states.

## Data Availability

All data is publically available through Google Trends and through The Behavioral Risk Factor Surveillance System (*BRFSS*).

## Abbreviations

**AIDS**: acquired immunodeficiency syndrome
**CDC**: Centers for Disease Control and Prevention
**CI**: confidence interval
**HIV**: *human immunodeficiency virus*
**PrEP**: pre-exposure prophylaxis
**SES**: socioeconomic
